# Complete genome sequence of *Atopobium parvulum* type strain (IPP 1246^T^)

**DOI:** 10.4056/sigs.29547

**Published:** 2009-09-23

**Authors:** Alex Copeland, Johannes Sikorski, Alla Lapidus, Matt Nolan, Tijana Glavina Del Rio, Susan Lucas, Feng Chen, Hope Tice, Sam Pitluck, Jan-Fang Cheng, Rüdiger Pukall, Olga Chertkov, Thomas Brettin, Cliff Han, John C. Detter, Cheryl Kuske, David Bruce, Lynne Goodwin, Natalia Ivanova, Konstantinos Mavromatis, Natalia Mikhailova, Amy Chen, Krishna Palaniappan, Patrick Chain, Manfred Rohde, Markus Göker, Jim Bristow, Jonathan A. Eisen, Victor Markowitz, Philip Hugenholtz, Nikos C. Kyrpides, Hans-Peter Klenk, John C. Detter

**Affiliations:** 1 DOE Joint Genome Institute, Walnut Creek, California, USA; 2 DSMZ - German Collection of Microorganisms and Cell Cultures GmbH, Braunschweig, Germany; 3 Los Alamos National Laboratory, Bioscience Division, Los Alamos, New Mexico, USA; 4 Biological Data Management and Technology Center, Lawrence Berkeley National Laboratory, Berkeley, California, USA; 5 Lawrence Livermore National Laboratory, Livermore, California, USA; 6 HZI - Helmholtz Centre for Infection Research, Braunschweig, Germany; 7 University of California Davis Genome Center, Davis, California, USA

**Keywords:** halitosis, obligately anaerobic, human respiratory tract, risk group 2, malodor, *Coriobacteriaceae*

## Abstract

*Atopobium parvulum* (Weinberg *et al.* 1937) Collins and Wallbanks 1993 comb. nov. is the type strain of the species and belongs to the genomically yet unstudied *Atopobium*/*Olsenella* branch of the family *Coriobacteriaceae*. The species *A. parvulum* is of interest because its members are frequently isolated from the human oral cavity and are found to be associated with halitosis (oral malodor) but not with periodontitis. Here we describe the features of this organism, together with the complete genome sequence, and annotation. This is the first complete genome sequence of the genus *Atopobium*, and the 1,543,805 bp long single replicon genome with its 1369 protein-coding and 49 RNA genes is part of the *** G****enomic* *** E****ncyclopedia of* *** B****acteria and* *** A****rchaea * project.

## Introduction

Strain IPP 1246^T^ (= DSM 20469 = ATCC 33793 = JCM 10300) is the type strain of the species *Atopobium parvulum* and was first described by Weinberg *et al.* 1937 as ‘*Streptococcus parvulus*’ (basonym) [1]. In 1992 it was reclassified as *A. parvulum* [2]. *A. parvulum* is of high interest because it has frequently been isolated from the human oral cavity, especially from the tongue dorsum, where it has been associated with patients suffering from halitosis (oral malodor) [3,4]. In general, the malodorous compounds are volatile sulfur compounds, with the most frequent ones being hydrogen sulfide, methyl mercaptan, and dimethyl sulfide, which are produced by bacterial metabolism of the sulfur containing amino acids cysteine and methionine [3,4]. However, for *A. parvulum* itself, the production of these substances has not yet been studied. *A. parvulum* has not been found to be significantly associated with chronic periodontitis, though a participation in periodontitis can not be fully excluded [5]. Nevertheless, *A. parvulum* has been associated with odontogenic infections, *e.g*. dental implants, but also with the saliva of healthy subjects [6]. Here we present a summary classification and a set of features for *A. parvulum* IPP 1246^T^ together with the description of the complete genomic sequencing and annotation.

## Classification and features

Phylotypes with significant 16S sequence similarity to strain IPP 1246^T^ were observed from intubated patients (EF510777) and from metagenomic human skin surveys (GQ081350) [7]. No significant similarities were found in human gut metagenomes (highest similarity is 92%, BABE01011651) [8], or in marine metagenomes (87%, AACY020304192) [9] (status June 2009).

**Figure 1:**
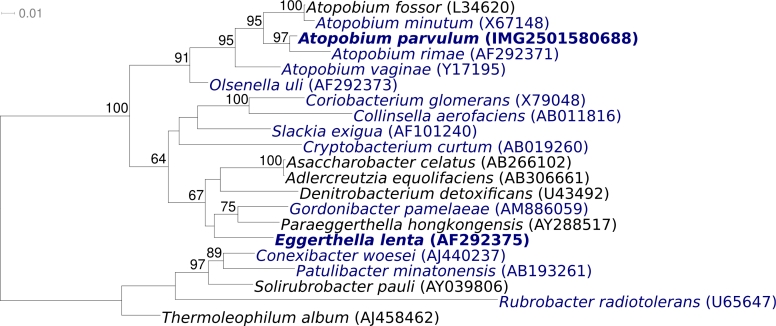
Phylogenetic tree of *A. parvulum* strain IPP 1246^T^, all other type strains of the genus *Atopobium* and the type strains of all other genera within the *Coriobacteriaceae*, inferred from 1345 aligned characters [10,11] of the 16S rRNA gene sequence under the maximum likelihood criterion [12]. The tree was rooted with the type strains of the genera within the subclass *Rubrobacteridae*. The branches are scaled in terms of the expected number of substitutions per site. Numbers above branches are support values from 1000 bootstrap replicates if larger than 60%. Lineages with type strain genome sequencing projects registered in GOLD [13] are shown in blue, published genomes in bold, including two of which are reported in this issue of *SIGS* [14,15]

Figure 1 shows the phylogenetic neighborhood of *A. parvulum* strain IPP P1246^T^ in a 16S rRNA based tree. The sequence of the sole copy of the 16S rRNA gene in the genome is identical with the previously published sequence generated from ATCC 22793 (AF292372), but differs by four nucleotides from the sequence used for the last taxonomic emendation (X67150) [2].

**Figure 2:**
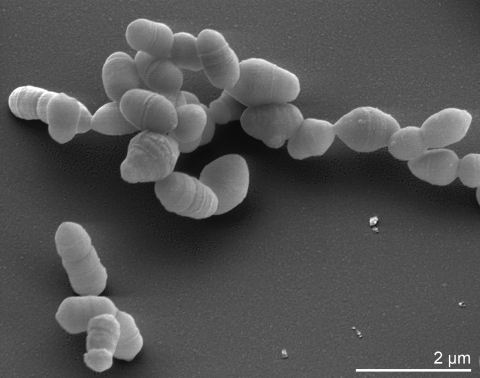
Scanning electron micrograph of *A. parvulum* IPP 1246^T^

The cells are cocci (approximately 0.3 to 0.6 µm in diameter) that occur singly, in pairs, in clumps, and in short chains, occasionally with central swelling [16,17] (Table 1 and Figure 2). The strains are non-motile and obligate anaerobic. Interestingly, growth is substantially stimulated by 0.02% (vol/vol) Tween 80 and by 10% (vol/vol) rabbit serum added to culture media [16]. Strain IPP 1246^T^ is susceptible to chloramphenicol (12 µg/ml), clindamycin (1.6 µg/ml), erythromycin (3 µg/ml), penicillin G (2 U/ml), and tetracycline (6 µg/ml) [17].

**Table 1:** Classification and general features of *A. parvulum* IPP 1146^T^ according to the MIGS recommendations [18].

**MIGS ID**	**Property**	**Term**	**Evidence code**
	Current classification	Domain *Bacteria*	TAS [19]
		Phylum *Actinobacteria*	TAS [20]
		Class *Actinobacteria*	TAS [20]
		Subclass *Coriobacteridae*	TAS [21]
		Order *Coriobacteriales*	TAS [21]
		Suborder “*Coriobacterineae”*	TAS [21]
		Family *Coriobacteriaceae*	TAS [21]
		Genus *Atopobium*	TAS [2]
		Species *Atopobium parvulum*	TAS [2]
		Type strain IPP 1246	
	Gram stain	positive	TAS [16]
	Cell shape	small cocci that occasionally appear to be elliptical	TAS [16]
	Motility	nonmotile	TAS [17]
	Sporulation	nonsporulating	TAS [16]
	Temperature range	25°C–45°C	TAS [17]
	Optimum temperature	37°C–45°C	TAS [17]
	Salinity	less than 6.5% NaCl	TAS [17]
MIGS-22	Oxygen requirement	obligate anaerobic	TAS [17]
	Carbon source	acid production from cellobiose, esculin, fructose, galactose, glucose, inulin, lactose, maltose, mannose, salicin, sucrose, and trehalose	TAS [17]
	Energy source	carbohydrates	TAS [17]
MIGS-6	Habitat	human respiratory tract.	TAS [1,17]
MIGS-15	Biotic relationship	free living	NAS
MIGS-14	Pathogenicity	associated with halitosis and human oral infections	TAS [3,4,6]
	Biosafety level	2	TAS [22]
	Isolation	unknown for this specific strain, but Weinberg *et al* reported that the principal habitat was the respiratory tract.	TAS [1,17]
MIGS-4	Geographic location	unknown, probably France	TAS [1,17]
MIGS-5	Sample collection time	before 1937	TAS [1,17]
MIGS-4.1 MIGS-4.2	Latitude – Longitude	unknown	
MIGS-4.3	Depth	not reported	
MIGS-4.4	Altitude	not reported	

Evidence codes - IDA: Inferred from Direct Assay (first time in publication); TAS: Traceable Author Statement (i.e., a direct report exists in the literature); NAS: Non-traceable Author Statement (i.e., not directly observed for the living, isolated sample, but based on a generally accepted property for the species, or anecdotal evidence). These evidence codes are from the Gene Ontology project [23]. If the evidence code is IDA the property was directly observed for a living isolate by one of the authors or another expert mentioned in the acknowledgements.

Strain IPP 1126^T^ produces acid (final pH < 4.7) from cellobiose, esculin, fructose, galactose, glucose, inulin, lactose, maltose, mannose, salicin, sucrose, and trehalose; erythritol and xylose were weakly fermented; no acid was produced from amygdalin, arabinose, glycerol, glycogen, inositol, mannitol, melezitose, melibiose, pectin, raffinose, rhamnose, ribose, sorbitol, or starch. Esculin was hydrolyzed; neither starch nor hippurate was hydrolyzed. Nitrate was not reduced. Indole was not formed. A solid acid curd formed in milk; neither milk, gelatin, nor meat was digested. Neither catalase, urease, deoxyribonuclease, lecithinase, nor lipase was detected [17]. Other enzyme activities are positive for acid phosphatase, alanine arylamidase, arginine arylamidase, β-galactosidase, leucine arylamidase, pyroglutamic acid arylamidase, glycine arylamidase, tyrosine arylamidase, but negative for arginine dihydrolase, histidine arylamidase, proline arylamidase, serine arylamidase, as determined using the API system [24].

### Chemotaxonomy

The chemotaxonomy of *A. parvulum* IPP 1246^T^ is unfortunately hardly studied. There are no data known on the polar lipids. The strain possesses cell-wall peptidoglycan of type A4α, L-Lys-D-Asp (type A11.31 according to the DSMZ catalogue of strains; http://www.dsmz.de/microorganisms/main.php?content_id=35) [25]. The major cellular fatty acids (FAME: fatty acid methyl ester; DMA: dimethylacetyl) are C_18:1_ *cis*-9 (38.2%, FAME), C_18:1_ *cis*-9 (24.1%, DMA), C_16:1_ *cis*-9 (5.0%, FAME), C_17:1_ *cis*-8 (5.0%, FAME), C_18:1_ c11/t9/t6 (5.0%, FAME), C_18:1_ *cis*-11 (3.9%, DMA), C_14:0_ (3.4%, FAME), C_10:0_ (3.0%, FAME) [16].

## Genome sequencing and annotation

### Genome project history

This organism was selected for sequencing on the basis of each phylogenetic position, and is part of the *** G****enomic* *** E****ncyclopedia of* *** B****acteria and* *** A****rchaea * project. The genome project is deposited in the Genome OnLine Database [13] and the complete genome sequence is deposited in GenBank Sequencing, finishing and annotation were performed by the DOE Joint Genome Institute (JGI). A summary of the project information is shown in Table 2.

**Table 2:** Genome sequencing project information

**MIGS ID**	**Property**	**Term**
MIGS-31	Finishing quality	Finished
MIGS-28	Libraries used	Two Sanger libraries: 8kb pMCL200 and fosmid pcc1Fos One 454 pyrosequence standard library
MIGS-29	Sequencing platforms	ABI3730, 454 GS FLX
MIGS-31.2	Sequencing coverage	7.8× Sanger; 43.4× pyrosequence
MIGS-30	Assemblers	Newbler, phrap
MIGS-32	Gene calling method	Prodigal, GenePRIMP
	Genbank ID	CP001721
	Genbank Date of Release	September 9, 2009
	GOLD ID	Gc01099
	NCBI project ID	29401
	Database: IMG-GEBA	2501533209
MIGS-13	Source material identifier	DSM 20469
	Project relevance	Tree of Life, GEBA

### Growth conditions and DNA isolation

*A. parvulum* strain IPP 1246^T^, DSM 20469, was grown anaerobically in DSMZ medium 104( modified PYG; Medium [26], ) at 37°C. DNA was isolated from 0.5-1 g of cell paste using the JGI CTAP procedure with a modified protocol for cell lysis as described in Wu *et al*. [27.

### Genome sequencing and assembly

The genome was sequenced using a combination of Sanger and 454 sequencing platforms. All general aspects of library construction and sequencing performed at the JGI can be found on the JGI website. 454 Pyrosequencing reads were assembled using the Newbler assembler version 1.1.02.15 (Roche). Large Newbler contigs were broken into 1,716 overlapping fragments of 1000bp and entered into assembly as pseudo-reads. The sequences were assigned quality scores based on Newbler consensus q-scores with modifications to account for overlap redundancy and to adjust inflated q-scores. A hybrid 454/Sanger assembly was made using the parallel phrap assembler (High Performance Software, LLC). Possible mis-assemblies were corrected with Dupfinisher [28] or transposon bombing of bridging clones (Epicentre Biotechnologies, Madison, WI). Gaps between contigs were closed by editing in Consed, custom primer walk or PCR amplification. A total of 125 Sanger finishing reads were produced to close gaps, to resolve repetitive regions, and to raise the quality of the finished sequence. The error rate of the completed genome sequence is less than 1 in 100,000. Together all sequence types provided 51.2 x coverage of the genome. The final assembly contains 12,842 Sanger and 359,479 pyrosequence reads.

### Genome annotation

Genes were identified using Prodigal [29] as part of the Oak Ridge National Laboratory genome annotation pipeline, followed by a round of manual curation using the JGI GenePRIMP pipeline [30]. The predicted CDSs were translated and used to search the National Center for Biotechnology Information (NCBI) nonredundant database, UniProt, TIGRFam, Pfam, PRIAM, KEGG, COG, and InterPro databases. Additional gene prediction analysis and functional annotation were performed within the Integrated Microbial Genomes Expert Review (IMG-ER) platform [31].

### Genome properties

**Figure 3:**
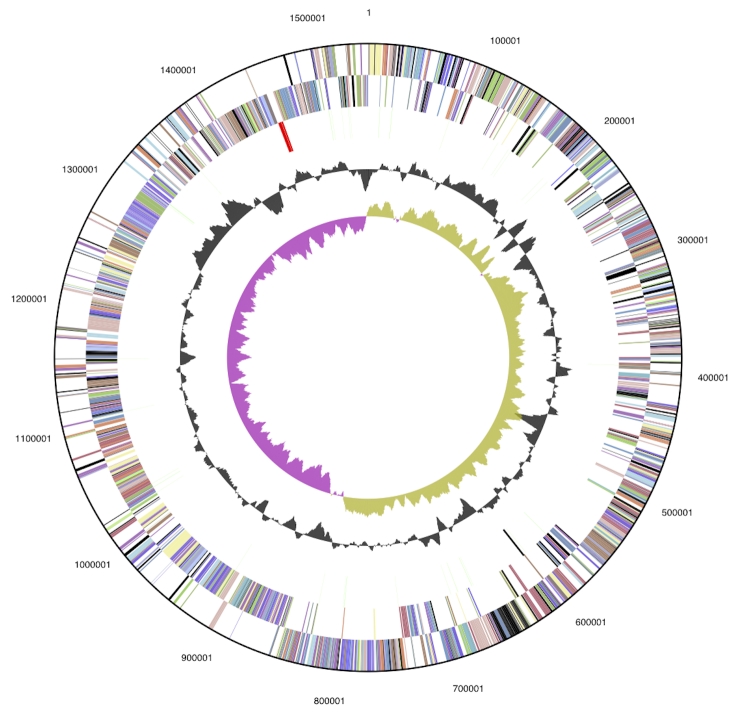
Graphical circular map of the genome. From outside to the center: Genes on forward strand (color by COG categories), Genes on reverse strand (color by COG categories), RNA genes (tRNAs green, rRNAs red, other RNAs black), GC content, GC skew.

The genome is 1,543,805 bp long and comprises one main circular chromosome with a 45.7% GC content (Table 3 and Figure 3). Of the 1419 genes predicted, 1369 were protein coding genes, and 50 RNAs. Sixteen pseudogenes were also identified. The majority of the genes (74.5%) were assigned with a putative function while the remaining ones were annotated as hypothetical proteins. The distribution of genes into COGs functional categories is presented in Table 4.

**Table 3:** Genome Statistics

**Attribute**	**Value**	**% of Total**
Genome size (bp)	1,543,805	100.00%
DNA Coding region (bp)	1,396,223	90.44%
DNA G+C content (bp)	705,312	45.69%
Number of replicons	1	
Extrachromosomal elements	0	
Total genes	1419	100.00%
RNA genes	49	3.52%
rRNA operons	1	
Protein-coding genes	1369	96.48%
Pseudo genes	16	1.13%
Genes with function prediction	1059	74.63%
Genes in paralog clusters	69	4.86%
Genes assigned to COGs	1096	77.24%
Genes assigned Pfam domains	1084	76.39%
Genes with signal peptides	240	16.91%
Genes with transmembrane helices	339	23.89%
CRISPR repeats	0	

**Table 4:** Number of genes associated with the general COG functional categories

**Code**	**Value**	**% age**	**Description**
J	128	9.3	Translation, ribosomal structure and biogenesis
A	0	0.0	RNA processing and modification
K	85	6.2	Transcription
L	72	5.3	Replication, recombination and repair
B	1	0.1	Chromatin structure and dynamics
D	18	1.3	Cell cycle control, mitosis and meiosis
Y	0	0.0	Nuclear structure
V	42	3.1	Defense mechanisms
T	46	3.4	Signal transduction mechanisms
M	70	5.1	Cell wall/membrane biogenesis
N	1	0.1	Cell motility
Z	0	0.0	Cytoskeleton
W	0	0.0	Extracellular structures
U	20	1.5	Intracellular trafficking and secretion
O	44	3.2	Posttranslational modification, protein turnover, chaperones
C	44	3.2	Energy production and conversion
G	115	8.4	Carbohydrate transport and metabolism
E	105	7.7	Amino acid transport and metabolism
F	53	3.9	Nucleotide transport and metabolism
H	37	2.7	Coenzyme transport and metabolism
I	23	1.7	Lipid transport and metabolism
P	59	4.3	Inorganic ion transport and metabolism
Q	11	0.8	Secondary metabolites biosynthesis, transport and catabolism
R	125	9.1	General function prediction only
S	90	6.6	Function unknown
-	273	19.9	Not in COGs
